# Nascent RNA transcripts facilitate the formation of G-quadruplexes

**DOI:** 10.1093/nar/gku416

**Published:** 2014-05-14

**Authors:** Prakash Shrestha, Shan Xiao, Soma Dhakal, Zheng Tan, Hanbin Mao

**Affiliations:** 1 Department of Chemistry and Biochemistry and School of Biomedical Sciences, Kent State University, Kent, OH 44242, USA; 2 State Key Laboratory of Biomembrane and Membrane Biotechnology, Institute of Zoology, Chinese Academy of Sciences, Beijing 100101, P.R. China

## Abstract

Recent discovery of the RNA/DNA hybrid G-quadruplexes (HQs) and their potential wide-spread occurrence in human genome during transcription have suggested a new and generic transcriptional control mechanism. The G-rich sequence in which HQ may form can coincide with that for DNA G-quadruplexes (GQs), which are well known to modulate transcriptions. Understanding the molecular interaction between HQ and GQ is, therefore, of pivotal importance to dissect the new mechanism for transcriptional regulation. Using a T7 transcription model, herein we found that GQ and HQ form in a natural sequence, (GGGGA)_4_, downstream of many transcription start sites. Using a newly-developed single-molecular stalled-transcription assay, we revealed that RNA transcripts helped to populate quadruplexes at the expense of duplexes. Among quadruplexes, HQ predominates GQ in population and mechanical stabilities, suggesting HQ may serve as a better mechanical block during transcription. The fact that HQ and GQ folded within tens of milliseconds in the presence of RNA transcripts provided justification for the co-transcriptional folding of these species. The catalytic role of RNA transcripts in the GQ formation was strongly suggested as the GQ folded >7 times slower without transcription. These results shed light on the possible synergistic effect of GQs and HQs on transcriptional controls.

## INTRODUCTION

Different deoxyribonucleic acid (DNA) species, such as various non-B DNA species and duplex DNA, form, dissolve and interconvert in the same genetic location. This highly dynamic population equilibrium closely resembles the population dynamics widely used in ecology to describe the change in the population of biological species due to processes such as birth, death, immigration and emigration (1). Inspired by this concept, recently, we developed a molecular population dynamics approach to decipher the equilibrium of non-B DNA and duplex DNA species (2). Dynamic pattern in population distribution offers flexibility for non-B DNA species to modulate cellular functions during different stages of a cell cycle. For example, it has been demonstrated that populations of DNA G-quadruplexes (GQs) show a periodic pattern, which correlates well with the activity of helicase known to dissolve these structures (3,4).

A GQ is a four-stranded structure formed by a stacking of planar G-quartets, which are linked through Hoogsteen hydrogen bonds. GQs have already been found in telomere and promoter regions *in vivo* (3) with proven biological functions such as transcription inhibitions (5). GQ-hosting sequences contain at least four guanine (G)-rich repeats. For GQ-hosting sequences with more than four G-rich repeats, different GQ units can form due to permutations of four G-rich repeats. Even for a sequence that hosts only one GQ unit, the conformation of the GQ can be surprisingly versatile (6,7). These scenarios bring complexity in the population equilibrium of GQ species. As prevailing species with long lifetime likely have significant biological roles, it becomes necessary to evaluate population profiles of GQs during various cellular processes.

Such an evaluation requires accurate determination of the population of individual species at equilibrium. In addition, as formation or dissolution of a species affects the overall equilibrium, evaluation of the transition kinetics among different species (8) becomes necessary. In single-stranded DNA regions such as telomere overhangs, the profiling of the molecular population dynamics is relatively simple. In double-stranded DNA (dsDNA) regions, however, non-B DNA structures in the complementary strand may participate in the dynamic equilibrium. During transcription, nascent ribonucleic acid (RNA) may contribute to the population dynamics of these non-B DNA structures as well. RNA is well known to fold into secondary or tertiary structures on its own or to form a hybrid DNA/RNA duplex in the presence of a complementary DNA strand (9–11). Recently, it has been suggested that the G-rich RNA transcript of the mitochondria CSB II motif can intermix with non-template DNA strand during transcription to form a new species, hybrid DNA/RNA G-quadruplex (HQ) (12). HQ formation was found to be a general phenomenon in transcription of G-rich DNA duplex (13,14). Sequences with the potential to form HQ are found to be evolutionally selected in the genomes of warm-blooded animals. They are preferentially located in the non-template DNA strand downstream of transcription start sites (TSSs) in >97% protein-encoding genes with an average of >73 sites per gene. The correlation of HQs with the transcriptional activity in host genes in human tissues has suggested their important roles in biological processes (13,14). Therefore, elucidating the effect of nascent RNA transcripts on the population dynamics that involve duplex DNA, various non-B DNA structures and DNA/RNA hybrid structures provides a new perspective, at the molecular level, to understand the biological functions of these species, especially for their roles in the transcriptional control.

However, the complex molecular population dynamics during transcription is rather difficult to investigate by ensemble methods, which can only resolve the structures and kinetics of pure species, but not the mixed populations. In ensemble methods, mutations are often required to populate one specific species over others to reveal its properties. This, however, disrupts the original population dynamics (7,15). Single-molecular techniques have an inherent capability to deconvolute individual species one molecule at a time and, therefore, present a superior solution to probe complex population dynamics. Among different single-molecule tools, force-based techniques, such as optical tweezers, can reveal spatial as well as mechanical information of DNA, RNA or protein structures (16–18).

In the research reported here, we evaluated the molecular interactions of GQ and HQ in the same G-rich location for the first time. We first employed ensemble approaches including DMS footprinting, ultraviolet (UV)-crosslinking and gel shift to investigate the formation of DNA GQ and DNA/RNA hybrid GQs in a naturally occurring G-rich region, 5′-(GGGGA)_4_, using a T7 transcription model. To delineate the kinetic and thermodynamic information of these quadruplex species, we introduced a single-molecule stalled-transcription assay (SMSA) in laser tweezers to unravel the details of molecular population dynamics hitherto unavailable to ensemble techniques. We observed a population equilibrium consisting of DNA GQ, partially folded DNA species and DNA/RNA HQs during transcription. HQ species consisting of nucleic acid strands with two G-rich repeats predominate among various populations. While HQ folds within 30 ms, the folding kinetics of DNA GQ is beyond the resolution of our force-jump approach (*τ* < 20 ms). The fast formation kinetics of quadruplexes suggests that co-transcriptional folding of GQ and HQ is possible. The folding of GQ is at least seven times slower (*τ* ∼ 150 ms) in the absence of the RNA transcripts, indicating the catalytic role of RNA to the formation of GQs. Not only HQs have higher population than GQs, but they are stronger in mechanical stability as well (unfolding force: 31 versus 25 pN), suggesting that HQs can potentially serve as stronger mechanical blocks to transcription. In addition, there is significant increase in the overall quadruplex population during transcription. All these findings suggest a synergistic effect of GQ and HQ to modulate transcription at the molecular level.

## MATERIALS AND METHODS

### *In vitro* transcription for ensemble experiments

A dsDNA construct containing a T7 promoter and a downstream G-core (5′-TT(GGGGA)_3_GGGGTT-3′) was prepared by overlap extension polymerase chain reaction. Transcription was carried out with 0.05-μM dsDNA at 37°C for 1 h in 20 μl of transcription buffer of 40-mM Tris-HCl (pH 8.0), 30% (w/v) Dimethyl sulfoxide (DMSO), 50-mM KCl, 2 U/μl of T7 RNA polymerase (Thermo Scientific, MA, USA), 8-mM MgCl, 10-mM Dithiothreitol (DTT), 2-mM spermidine, 2-mM adenosine triphosphate (ATP), Uridine triphosphate (UTP) and Guanosine monophosphate (GMP), 1-mM Guanosine triphosphate (GTP) or 7-deaza-GTP and 0.005 U/μl inorganic pyrophosphatase, (Thermo Scientific, MA, USA). An equal volume of buffer containing 30% DMSO, 50-mM KCl, 1 μM of competitive DNA and the indicated RNases (0.8-μg/μl RNase A, 0.4-U/μl RNase H) was added and the mixture was maintained at 37°C for 1 h to terminate the transcription and digest the RNA. DNA samples were then resolved at 4°C, 8 V/cm on a 10% polyacrylamide gel in 1× Tris-borate-ethylenediaminetetraacetic acid (EDTA) buffer that contained 75-mM KCl (13,19) and DNA detected by the fluorescence of carboxyfluorescein (FAM) dye labeled at the 5′ end of the non-template strand using a Typhoon 9400 phosphor imager (GE Healthcare, PA, USA).

### Analysis of the RNA transcript

Transcription was carried out as aforementioned but with addition of 0.05-mM fluoresein-12-UTP (Roche, Switzerland). The samples were digested with 0.08 U/μl of DNase I (Thermo Scientific, MA, USA) at 37°C for 30 min. The reaction was stopped by adding an equal volume of 40-mM EDTA. The samples were extracted with equal volume of phenol/chloroform (1/1, v/v), dissolved in 50% deionized formamide and resolved on an 8% denaturing gel.

### DMS footprinting

Transcribed DNA (100 μl) was mixed with an equal volume of Tris-HCl (pH 7.9) buffer containing 30% (w/v) DMSO, 50-mM KCl, 40-mM EDTA and 0.2% sodium dodecyl sulphate. The DNA was then subjected to footprinting as described (20).

### UV-crosslinking

Transcription was carried out as aforementioned but with the normal UTP being substituted with 1 mM of 4-S-UTP (TriLink BioTechnologies, CA, USA). Hundred microliters of transcribed and RNase H-digested DNA was subjected to UV-crosslinking as described (13,19). The DNA was then treated by RNase A and EDTA as aforementioned, recovered with phenol/chloroform extraction and purified by the TIANquick mini purification kit (Tiangen, Beijing). Primer extension was performed with 0.4-μM 5′-FAM-CCAGCCTGCGGCGAGTG primer, 4 U of Deep VentR DNA polymerase (exo-) (NEB, MA, USA) in a 50-μl volume containing 75-mM Tris-HCl, pH 8.8, 20-mM (NH_4_)_2_SO_4_, 0.1% (v/v) Tween 20, 2-mM MgCl_2_, 0.05-mM dNTP and 5% (v/v) DMSO. G and T ladders were synthesized in the same way using a non-transcribed and non-crosslinked DNA strand in the presence of Acy-CTP and Acy-ATP (NEB), respectively, in a molar ratio of 1/2 and 1/1 to dCTP and dATP, respectively.

### Detection of RNase-resistant RNA in transcribed DNA

Transcription was conducted with DNA amplified using a biotinylated downstream primer. Fifty microliters of streptavidin-coated MagneSphere paramagnetic particles (Promega, WI, USA) was washed three times, each with 2× volume of 10-mM Tris-HCl buffer (pH 8.3) containing 30% DMSO, 50-mM KCl and 10-ng/μl fish sperm DNA. They were then incubated with 100 μl of transcribed DNA pre-digested with RNase A and H for 15 min at 37°C. After three times wash each with a 500 μl of 10-mM Tris-HCl buffer (pH 8.3) that contains 30% DMSO, 50-mM KCl, 10-ng/μl fish sperm DNA and 20-mM EDTA, the immobilized DNA was detached by heating at 95°C for 10 min in 30 μl of 20-mM EDTA and then mixed with an oligomer probe (5′-A(CCCCT)_3_CCCCA-3′) that was labeled with a Cy5 at the 5′ end. The samples were cooled down slowly and resolved on 10% native polyacrylamide gel electrophoresis (PAGE) gel at room temperature.

### Preparation of DNA construct for single-molecule assays

The DNA oligomers purchased from Integrated DNA Technologies (www.idtdna.com) were further purified by denaturing PAGE gel and stored at −20°C. The polystyrene beads coated with streptavidin or anti-digoxigenin for the single-molecule experiments were purchased from Spherotech (Lake Forest, IL, USA).

DNA constructs for the single-molecule assay were synthesized according to the flow chart described in Supplementary Figure S2. First, dsDNA containing a T7-promoter site (5′-TAA TAC GAC TCA CTA TA), a G-core (see above) and a stall site downstream of the G-core was prepared by melting two complementary strands at equimolar ratio at 95°C for 10 min, followed by slow cooling to 25°C in 6 h. One end of the dsDNA contains an XbaI restriction site and the other an EagI site so that this DNA fragment can be ligated between two long dsDNA handles (2028 bp derived from pBR322 and 2690 bp derived from pEGFP) with respective restriction sites. For effective ligation, molar ratio of 4:1:4 (handle:dsDNA-Construct:handle) was used. The free ends of the 2028 and 2690-bp handles were labeled with biotin and digoxigenin, respectively. In single-molecule experiments, these two ends were tethered to the two optically trapped beads coated with streptavidin and anti-digoxigenin antibody, respectively. The DNA construct with two stall sites (see Supplementary Figure S3) was prepared similarly.

### Preparation of nucleic acid constructs with stall transcriptions

Regular transcription was carried out by mixing 1-μl T7 RNAP (NEB, 50 000 U/ml) with 1 μl of the dsDNA construct (see the construct for one stall site in Supplementary Figure S3) prepared above in a pH 7.8 transcription buffer (40-mM Tris-HCl, 6-mM MgCl_2_, 10-mM dithiothreitol, 2-mM spermidine in 30% DMSO solution). After mixing with three nucleotide triphosphates (NTPs) (0.5 mM each of ATP, GTP and UTP), 10-μl reaction mixture was incubated at 37°C for 15 min to transcribe the DNA and to stall the T7 RNAP at the cytosine site (see Supplementary Figure S3). During the deaza transcription, all conditions were the same as the regular transcription except GTP was replaced by 7-deaza-GTP. During the transcription of the DNA template with the 2-stall sites, T7 RNAP was stalled at the first site (see Supplementary Figure S3) by supplying ATP and GTP for 10 min. Extra T7 RNAP was dissociated from 1.5-μM DNA with a competitive promoter sequence, 5′-GAA ATT AAT ACG ACT CAC TAT A. A mixture of 0.5 mM each of ATP, GTP and UTP was then added for 15 min to allow the RNAP to stall at the second stall site. This procedure should allow only one passage of the RNAP over the G-core region.

### Mechanical unfolding in laser tweezers

The laser-tweezers setup for the mechanical pulling experiments has been described previously (16,21). Briefly, a diode pumped solid state laser (1064 nm, 4 W, Continuous-wave (CW) mode, BL-106C, Spectra-physics) was used to form two optical traps. One trap was controlled by a steerable mirror (Nano-MTA, Mad City Laboratories) at a conjugate plane of the back focal plane of a focusing objective (Nikon CFI-Plan-Apochromat 60×, NA 1.2, water immersion, working distance ∼320 μm). The exiting two beams were collected by an identical objective and detected separately by two position sensitive photodetectors (PSD, DL100, Pacific Silicon Sensor) (22).

Unless otherwise specified, all pulling experiments were carried out at 23°C in the transcription buffer with 30% DMSO as described above. The DNA construct with stalled T7 RNAP prepared above was immobilized onto a 2.10-μm bead via digoxigenin–anti-digoxigenin antibody interaction. The mixture was then diluted to 1-ml transcription buffer and injected into a microfluidic chamber. To prevent subsequent binding and transcription of other RNAPs, the chamber was filled with the transcription buffer containing 30% DMSO but without T7 RNAP. After trapping the DNA-immobilized bead and the streptavidin-coated bead by two separate laser foci, the DNA construct was tethered between these two beads. One of the beads was then moved away from the other, increasing the tension inside the DNA construct until structure was unfolded. In a typical force–extension experiment, the tethered DNA was extended below the plateau force (maximum 60 pN) and relaxed to 0 pN with a loading rate of 5.5 pN/s. The kinetic measurement was carried out by the force pumping and probing (FPP) approach (8,23).

### Data analyses for SMSAs

The raw data were recorded at 1000 Hz in a LabVIEW program (National Instruments, Austin, TX, USA) and Savitzky-Golay filtered to 100 Hz by MATLAB (The Math Works, Natick, MA, USA), followed by analyses using IGOR programs (WaveMetrics, Portland, OR, USA). The rupture force (*F*_rupture_) was measured directly from the force–extension (*F–X*) curves, and the change in contour length (Δ*L*) was calculated from the two data points flanking the rupture event. The Δ*L* was also measured by the following equation derived from the worm-like-chain (WLC) model (24),

(1) \begin{equation*} \frac{{\Delta x}}{{\Delta L}} = 1 - \frac{1}{2}\left( {\frac{{k_b T}}{{FP}}} \right)^{1/2} + \frac{F}{S}, \end{equation*}

where Δ*x* is the difference in extension between the stretching and relaxing curves at a particular force (*F*), *k*_b_ is the Boltzmann constant, *T* is the absolute temperature, *P* is the persistent length (50.9 ± 1.6 nm) (25) and *S* is the stretching modulus (1168 ± 119 pN) (25). Both Δ*L* measurements yielded identical values.

The kernel density calculation and bootstrapping analysis of Δ*L* [*Po*pulation *D*econvolution at *Nano*meter (PoDNano) analyses] were carried out as described in literature (26). The Δ*L* populations were identified by 3000 times in resampling. Percent formation for different populations was estimated from the *F–X* curves that contain the unfolding of specific Δ*L* species versus the total *F–X* curves (see Supplementary Information for details). For two populations that are closely located with an intersection region, random deconvolution was performed as described (27).

## RESULTS AND DISCUSSION

### Ensemble experiments show the formation of HQ species as a result of transcription

We chose a natural sequence, 5′-(GGGGA)_4_ (G-core), as a model system to probe the complex equilibrium of G-quadruplexes during the transcription catalyzed by T7 RNAP. By genomic analyses, this sequence has been found in the non-template strand of 158 genes (Supplementary Table S1 shows 36 genes that contain the G-core within 10 000 bp downstream of the TSS). Since this sequence is located downstream of the TSS, RNA strands containing the same G-core sequence will be produced during transcription. These RNA strands may participate in the equilibrium in which GQ could form.

**Figure 1.**
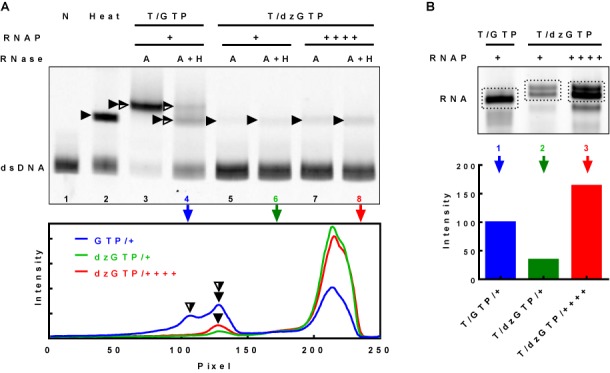
(**A**) Detection of G-quadruplex formation by native gel electrophoresis in G-rich dsDNA with GTP or 7-deaza-GTP (dzGTP) and two other NTPs followed by digestion with RNase A or RNase A+H. RNase A can digest ssRNA, RNase (A+H) can digest R-loop, while HQ is resistant to either RNase A or H. The bottom panel shows intensity scan of gel lanes 4, 6 and 8. Filled arrowheads indicate intramolecular GQ and half-filled arrowheads indicate HQ. (**B**) Transcription efficiency in the presence of normal GTP or dzGTP. RNA product was resolved on a denaturing gel without RNase digestion. The bottom graph shows the quantification of the RNA (dotted box) in the three lanes of the gel. ‘N’ stands for no transcription; ‘Heat’ stands for denaturation/renaturation; ‘T/GTP’ and ‘T/dzGTP’ stand for transcription with GTP and dzGTP, respectively. ++++ indicates four folds of RNA polymerase (RNAP) added comparing to +.

Formation of the GQ was first detected by native gel electrophoresis. It has been shown that DNA containing G-quadruplexes should have a slower migration than the dsDNA counterpart (13,14). Therefore, the slower moving band in the gel (Figure 1A, lane 2, filled arrowhead) suggested the formation of an intramolecular G-quadruplex in the DNA that contains four G-tracts after the molecule was subjected to a heat denaturation/renaturation cycle. To detect G-quadruplex formations in the transcribed DNA, samples were treated with RNases A and H either separately or in combination. RNA in HQ is resistant to both RNases (13,19). The DNA transcribed in a K^+^ buffer with regular GTP showed an extra band after digestion with RNase A (Figure 1A, lane 3, full and half-filled arrowhead), which was more retarded than the DNA carrying an intramolecular G-quadruplex (Figure 1A, lane 2). Transcription of G-rich DNA results in the formation of R-loops in which a nascent RNA transcript is base-paired with the template DNA at the G-rich region (28). Because RNase A only cleaves at the 3′ end of cytidines and uridines in single-stranded RNA (ssRNA) or RNA in the DNA/RNA duplex at low salt concentrations (0–100 mM) (13), the G-core region of the RNA (either in hybrid G-quadruplexes or RNA loops) is resistant to RNase A. Therefore, the slower migration of this extra band can be explained by a structure containing G-quadruplex, R-loop or both. To validate this assignment, transcribed product was treated with RNases A and H to cleave both *ss*RNA and R-loop (Figure 1A, lane 4) (19). It was found that the amount of the fully-annealed DNA was increased (Figure 1A, lane 4, bottom band), suggesting the presence of the R-loop structure in the slow-migrating band in lane 3. The migration of a smaller portion of the DNA remained unchanged (lane 4, half-filled arrowhead), suggesting a fragment of RNA was protected from the digestion of RNases A and H, probably due to the formation of HQ structures. When the RNA was prevented from forming G-quadruplex in transcription by substituting regular GTP with dzGTP which lacks the 7′-nitrogen for G-quartet assembly, this band disappeared (lane 5). This result suggests that the slowest band represented HQ structure. On the other hand, a significant portion of the slow-migrating DNA in lane 3 restored the mobility (lane 4, middle band) to the same level as the DNA containing the intramolecular G-quadruplex in the heated sample (lane 2). We assume that this band contained a different species of HQ as well as GQ. The former structure seemed to be the major structure since this band was significantly reduced when the transcription was carried out with dzGTP, in which only GQ could be produced (lanes 6 and 8).

dzGTP reduces the incorporation at the initial nucleotide (29), which can be restored by addition of GMP (13,19,29). To avoid this problem, therefore, all our transcriptions with dzGTP were supplied with GMP. However, the transcript analysis in Figure 1B indicated that the dzGTP still showed a significant reduction in the transcription efficiency (lane 2 versus lane 1). To ensure the changes observed with the GTP substitution was not due to reduced transcription efficiency, we increased the amount of T7 polymerase by 4-folds, which elevated the transcription efficiency to 160% (lane 3 versus lane 1). This treatment, however, did not alter the result that HQ did not form in the presence of dzGTP (Figure 1A, bottom graph). These results confirmed that the HQ was RNA dependent.

To confirm that the G-core fragment can form HQ with an RNA strand of the same sequence, we hybridized an RNA oligonucleotide with a DNA fragment (DNA1) to form a partial duplex construct (Supplementary Figure S1A). Both oligomers carried an overhanging G-core that could possibly form an HQ. Another DNA:RNA partial duplex construct consisting of a mutated G-core DNA (DNA2) was used as reference. The HQ formation was detected by native gel electrophoresis after the RNA component in the duplex stem region was digested by RNase A or H. The much slower migration of the bands in RNA/DNA1 with respect to RNA/DNA2 can be ascribed to the association of the RNA G-core with the DNA1 via the HQ formation. The formation of HQ in the RNA and DNA1 was further confirmed by Circular Dichroism (CD) spectroscopy. A negative peak near 245 nm and a positive peak near 265 nm (Supplementary Figure S1B) are characteristic of a parallel G-quadruplex and similar to those of the HQ we recently reported (19).

**Figure 2.**
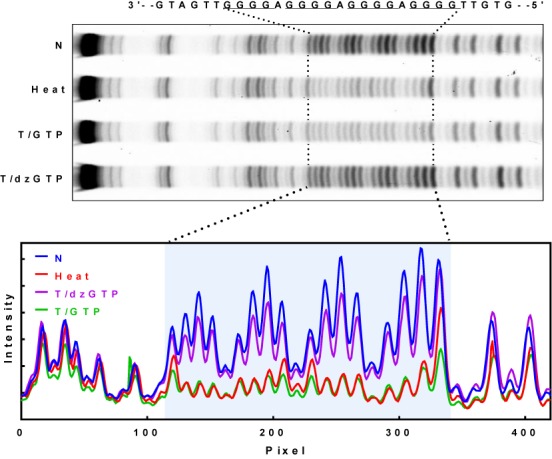
Participation of the non-template DNA strand in HQ formation detected by DMS footprinting. DNA was not transcribed (NT), heated (Heat) without transcription and was transcribed with GTP (T/GTP) or dzGTP (T/dzGTP). DNA cleavage fragments were resolved on a denaturing gel.

Next, we examined the participation of DNA in the G-quadruplexes by DMS footprinting in which guanine residues assembled into a G-quadruplex are protected from chemical cleavage (30). In Figure 2, formation of G-quadruplex in the heated and transcribed DNA led to obvious protection of the guanines in the G-core. In the transcribed DNA, all the four G-tracts were similarly protected as those in the lane with heating. This could imply a similar probability for each G-tract to participate in the HQ assembly. In contrast, the DNA transcribed with the 7-deaza-GTP showed much reduced protection, further supporting the formation of HQ in transcription.

**Figure 3.**
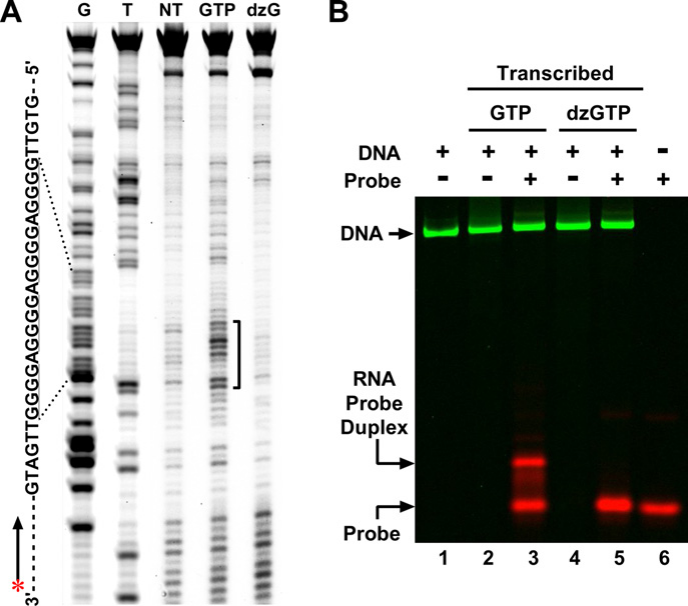
(**A**) UV-crosslinking to show the participation of G-tracts of RNA in G-quadruplex formation. 4-thio-UTP was incorporated into the RNA transcript and subjected to a post-transcription UV irradiation. Crosslinking between RNA and DNA was detected by primer extension on the non-template DNA strand. Lanes from left to right represent G ladder, T ladder, DNA without transcription and DNA transcribed with GTP or dzGTP, respectively. (**B**) Detection of the RNase-resistant RNA fragments in HQ. DNA transcribed with GTP or dzGTP was immobilized on magnetic beads and treated with RNase A and H. After washing, the RNA G-core was released and hybridized with a Cy5-labeled oligomer probe and resolved on a native gel.

In our previous work, we used UV-crosslinking to verify the participation of RNA in the HQ (13,19). For the DNA used here in which the G-core is able to form an intramolecular GQ, it is especially important to prove the involvement of the RNA transcript. To this purpose, we introduced a di-thymidine (TT) sequence at both 5′ and 3′ sides of the G-core region in the non-template DNA strand. During transcription, we used 4-thio-UTP instead of UTP to incorporate modified uridines near the G-core. Due to the possible HQ assembly, the thio-modified uridines would covalently crosslink with nearby nucleotides in the non-template DNA strand upon UV irradiation. In Figure 3A, crosslinking was detected at the two G-tracts from the 3′ end of the G-core (GTP lane). The involvement of the hybrid G-quadruplex structure to the crosslinking was obvious as the crosslinked bands disappeared when transcription was carried out using 7-deaza-GTP (dzG lane) instead of GTP.

To further verify the participation of RNA in the HQ assembly, we immobilized transcribed DNA modified with biotin on streptavidin-coated magnetic beads after it was treated with RNases A and H. As described previously, these two enzymes can digest the RNA in the single-stranded form or hybridized with the template DNA, but not the RNA in the hybrid G-quadruplex. After washing, the RNA was released from the beads and probed with a Cy5-labeled DNA oligomer complementary to the RNA G-core (see the Materials and Methods section). In Figure 3B, RNA G-core was detected as indicated by an extra probe band above the original probe (lane 3). When the transcription was conducted with 7-deaza-GTP to prevent HQ formation, this extra band almost disappeared, strongly suggesting that the RNA in the band was associated with the HQ (lane 5).

Taken together, the gel-shift, DMS footprinting and photo-crosslinking experiments provided convincing evidence for the formation of R-loop, GQs and DNA/RNA hybrid G-quadruplexes during transcription.

### SMSA confirms transcription-induced DNA/RNA hybrid G-quadruplex species

**Figure 4.**
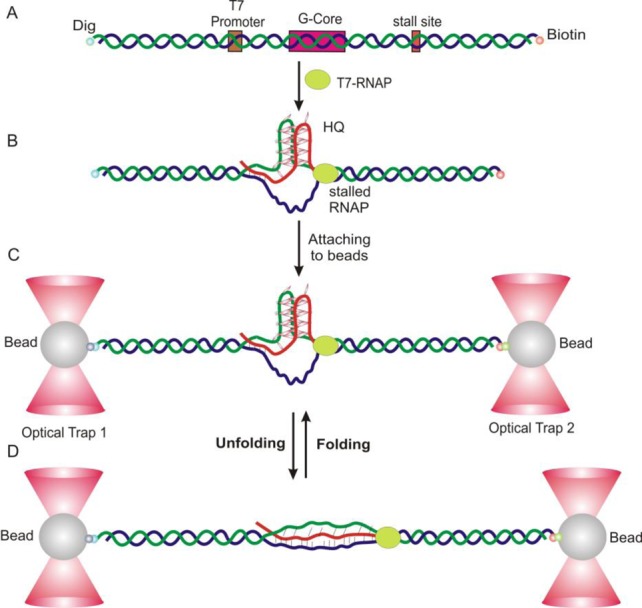
Schematic of the SMSA in optical tweezers. (**A**) The DNA construct. G-core has a sequence of 5′-(GGGGA)_4_. (**B**) The T7 RNAP is stalled during a transcription carried out in 30% DMSO (pH 7.4). (**C**) DNA construct with stalled RNAP is tethered between two optically trapped beads. (**D**) Mechanical unfolding of structures formed in the G-core during transcription.

To confirm the presence of GQ and HQ found in ensemble assays, we proceeded with mechanical unfolding approaches. As T7-RNA polymerase covers ∼10 bases during transcription (31), we designed a stall site 15 nucleotides (nts) downstream of the last G in the G-core to avoid the interference of the polymerase on the formation of GQ (Supplementary Figure S3). The stalled transcription complex was tethered between two optically trapped beads for mechanical unfolding and refolding experiments (see Figure 4 and the Materials and Methods section) (32).

**Figure 5.**
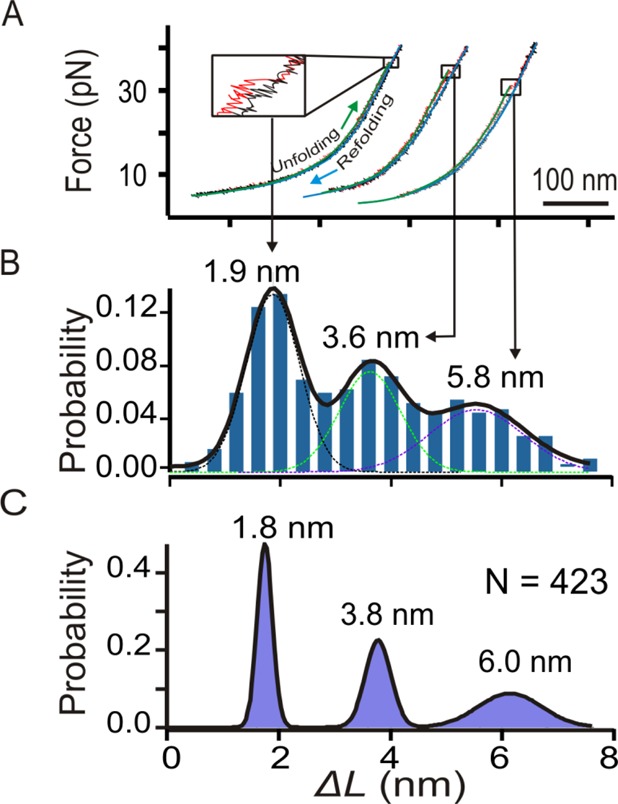
(**A**) Typical *F–X* curves show the unfolding transitions of three different species with Δ*L* ∼1.9 nm, 3.6 nm and 6.0 nm during regular stalled transcription. Each curve is fit by the WLC model for stretching (green) and relaxing (blue) processes. (**B**) Histogram of the change in contour length (Δ*L*) measured at the rupture force. Solid curves depict 3-peak Gaussian fitting and dotted curves represent Gaussian fittings for individual populations. (**C**) Histogram of Δ*L* populations after the PoDNano treatment. Black curves depict Gaussian fittings.

When the two beads were moved apart, the tension in the nucleic acid construct increased, which led to the unfolding of the structures as indicated by a sudden change in force or extension in a force–extension (*F–X*) curve (see Figure 5A). After each unfolding, tension in the DNA construct can be brought to zero at which the unfolded species starts to refold while both RNA transcript and RNAP stay in close proximity. Therefore, the setup closely mimics the co-transcriptional folding process. As repetitive unfolding and refolding of nucleic acid structures can be carried out readily without requirement of new passages of RNAP, such a setup significantly increases the throughput of single-molecular experiments.

Typical *F–X* curves during regular stalled transcription showed three different types of unfolding events measured by the change in contour length (Δ*L*) (Figure 5A). In the Δ*L* histograms shown in Figure 5B, three populations with Δ*L* = 1.8 ± 0.2, 3.8 ± 0.5 and 6.0 ± 0.6 nm are obvious. PoDNano resolution (26) using resampling and bootstrap strategy confirmed the presence of the three populations (Figure 5C). Calculation revealed that these three structures contain 9, 14 and 19 nts, respectively (see Supplementary Information for calculation). While the 19-nt population corresponds to a fragment that contains four GGGGA tracts, the 14- and 9-nt species contain 3 and 2 GGGGA tracts, respectively. Inspired by the ensemble assays in which hybrid DNA/RNA GQs (HQ) were observed, we propose that populations with 9 and 14 nts (1.8 and 3.8 nm in Δ*L*, respectively) are likely HQ species that consist of two and three G-tracts from the non-template DNA in combination with two and one G-tract of RNA transcripts, respectively (2G-HQ and 3G-HQ).

To confirm these assignments, four control experiments were performed. In the first control, we mutated guanine bases in the G-core to obtain a sequence, 5′-GTT GAT TAG ATG TGA TTA G. Since this sequence lacks consecutive guanine residues, neither GQ nor HQ is expected to form. When the sequence is subject to the mechanical unfolding with or without transcription, indeed, we observed very few unfolding events with Δ*L* ranging from 2 to 8 nm (Supplementary Figure S4). This result indicates that all three major populations observed in Figure 5A are structures associated with G-tracts. Next, we performed mechanical unfolding of the 5′-(GGGGA)_4_ in the presence of 100-mM Li^+^ after T7 RNAP was stalled during transcription. As expected, we observed only a few folded structures (6.7%; Supplementary Figure S5). It has been shown that the formation of G-quadruplexes is much inhibited in Li^+^ (33); therefore, the result in Supplementary Figure S5 supports our assignment of GQ and HQ species observed in Figure 5.

**Figure 6.**
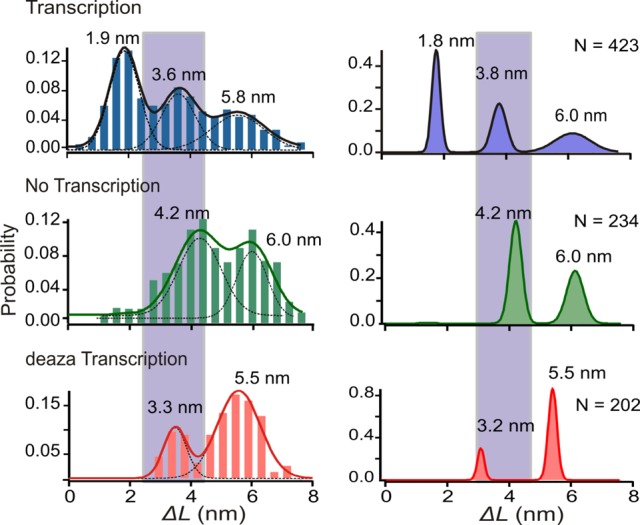
Population distribution of species with regular transcription (top panel), without transcription (middle panel) and with transcription in the presence of 7-deaza-GTP (bottom panel). Left panels depict regular Δ*L* histograms measured at the rupture force while right panels show Δ*L* histograms after the PoDNano treatment. Curves represent Gaussian fittings.

In the third control, mechanical unfolding of the 5′-(GGGGA)_4_ DNA construct was performed without transcription. As clearly shown in Figure 6 (middle panel), the smallest Δ*L* population disappeared while the remaining two populations showed slightly larger Δ*L* values (Δ*L* ∼ 4.2 and 6.0 nm). While the largest Δ*L* population represents GQ, the ∼4.2-nm Δ*L* population may represent partially folded structures, such as G-triplex (GT), as observed previously in other G-quadruplex forming sequences (34). This control indicated that the ∼2.0-nm Δ*L* species observed in Figure 5B and C should involve RNA strands while the 3.6-nm Δ*L* (Figure 5) population can be a GT or an HQ. In the last control, we transcribed the same DNA construct by using 7-deaza-GTP instead of GTP. The 7-deaza guanine in RNA is known to compromise Hoogsteen hydrogen bonding and therefore HQ is not expected to form (13). The Δ*L* histogram clearly revealed a major population at 5.5 ± 0.2 nm, a minor population at 3.3 ± 0.4 nm, and the ∼2.0-nm species was again absent (Figure 6, bottom panel). Similar to the third control, this result indicated that the ∼2.0-nm population in Figure 5B and C should be an HQ species while the 3.3-nm species could be a partially folded DNA structure such as GT.

Since the ∼2-nm species only involves 9 DNA nucleotides that span two GGGG tracts, it is clear that this species is an HQ that employs two DNA G-tracts and two RNA G-tracts (or 2G-HQ). However, for the ∼4-nm and ∼6-nm species in Figure 5B and C, they may represent HQs that employ at least a total of three DNA G-tracts with at least one GGGG tract serving as an internal loop. To test whether non-tandem G-tracts can be employed in HQ, we prepared a construct that consists of two G-tracts interspersed by a 6-nt spacer, ATTTTA. Mechanical unfolding experiments under the stalled transcription condition revealed only little formation (7.9%) of structures with 2–8 nm in Δ*L* (Supplementary Figure S6), strongly suggesting that consecutive G-tracts are required to form DNA/RNA hybrid G-quadruplexes.

Such a result indicates that the ∼6-nm species observed during the regular transcription can be a GQ or two neighboring 2G-HQs, while the ∼4-nm population can be a partially folded DNA GT or an HQ that employs three consecutive DNA G-tracts with one RNA G-tract (3G-HQ). During the unfolding, however, we did not observe two consecutive events that correspond to the unfolding of two tandem 2G-HQ species. This observation led us to propose that the ∼6-nm species should be a DNA GQ. To estimate the ratio of GT and HQ in the ∼4-nm population, we first calculated the ratio of GT and GQ in the 7-deaza-GTP transcription in which no HQ should be present. We rationalized that GT and GQ maintain the same ratio based on the fact that both 7-deaza-GTP and regular transcriptions were carried out under the same set of conditions. Using this ratio, we can then estimate the amount of GT in the ∼4-nm population by measuring the GQ population in the ∼6-nm peak (Figure 6, top panel). This algorithm gave the percentage formation of 9.4% and 3.8% for HQ and GT species, respectively, in the ∼4-nm population (Figure 6, top panel; see Supplementary Information for calculation).

### Population dynamics of the HQ and GQ species

#### Population distribution of the GQ and HQ species

**Figure 7.**
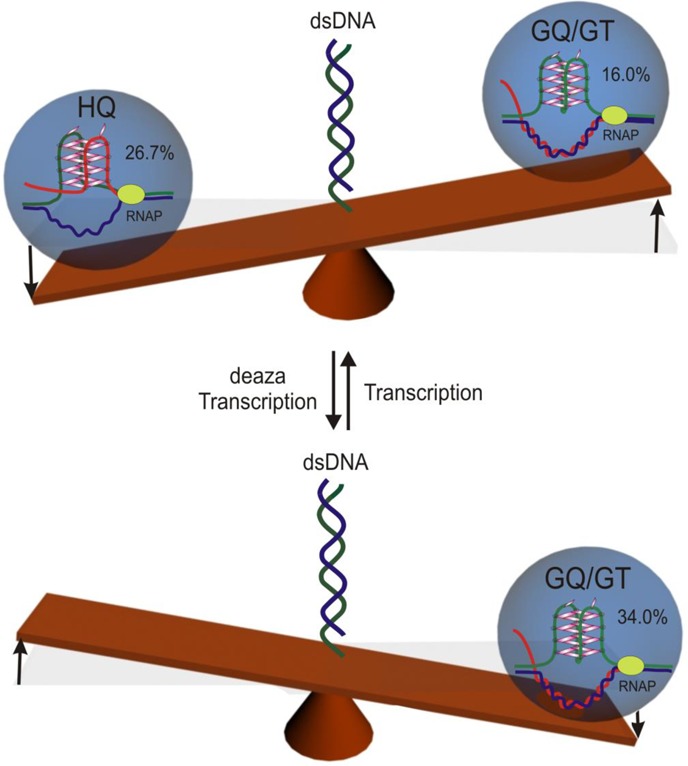
Schematic representation of population dynamics of DNA G-quadruplex (GQ), partially folded DNA species (GT) and DNA/RNA hybrid G-quadruplex (HQ) formed during the regular transcription (top) and transcription in the presence of 7-deaza-GTP (deaza transcription; bottom).

With a clear assignment of individual species, next, we compared population percentages of GQ, HQ and dsDNA species in different transcription experiments (Table 1; see Supplementary Information for detailed calculation). First, we found that transcription almost doubled the combined population of HQ and GQ/GT with respect to that without transcription (43% versus 24%; see Table 1). This surprising result indicated the importance of RNA transcripts for the formation of HQ and GQ species. Population comparison during regular transcription also revealed that the HQ species predominated over the GQ/GT species (26.7% versus 16.0%; see Figure 7 and Table 1), suggesting a more significant role for the HQ in gene expressions. Notably, the population estimation in single-molecule experiments (57.3% for dsDNA; Figure 7 and Table 1) matched closely with that obtained from ensemble gel shift assays (62.7% in Figure 1A, lane 4), which demonstrates the accuracy of the single-molecule measurements.

**Table 1.** Percent formation of different populations during transcription, transcription with 7-deaza-GTP and no transcription

Δ*L* (nm)	% Formation		
	Transcription	deaza-GTP transcription	No transcription
1.8 (2G HQ)	17.3	0	0
3.6 (3G HQ/GT)	13.2 (9.4/3.8)	8.1 (0/8.1)	14.7 (0/14.7)
5.5 (GQ)	12.2	25.9	9.6
0 (dsDNA or R-loop)	57.3	66	75.7

#### Mechanical and thermodynamic stabilities of the GQ and HQ species

**Figure 8.**
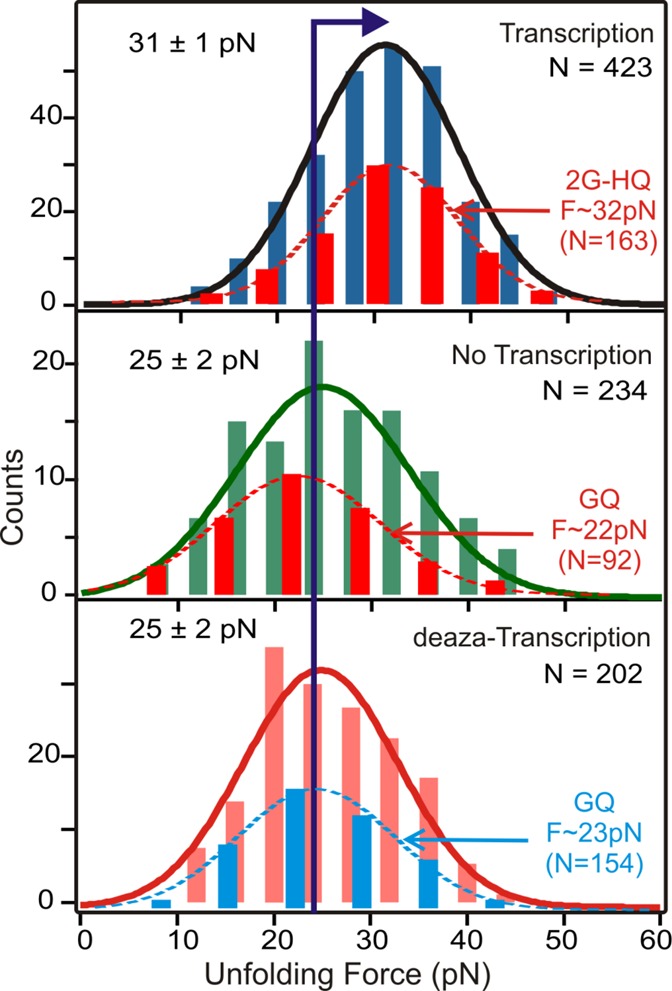
Rupture force histograms observed during regular transcription (top panel), no transcription (middle panel) and transcription with 7-deaza GTP (bottom panel). The dotted histogram in each panel depicts the rupture force of HQ or GQ population after deconvolution (see text). For clarity, these dotted histograms are plotted with reduced size.

Recent evidence in our lab has suggested that the HQ species formed during the first few rounds of transcription could stall subsequent transcription processes (19). Since RNAP that catalyzes transcription is a motor protein which possesses certain load force, the unfolding force of HQs could be used to evaluate whether HQ can serve as a mechanical block to the RNAP by comparing with the stall force of RNAPs (35,36). The force-based single-molecule experiment employed here has a unique ability to determine the mechanical stability of folded species (37). The histograms of the unfolding force measured by the single-molecule transcription assay are summarized in Figure 8. While the unfolding force of the species formed during transcription is centered at ∼31 ± 1 pN (it is ∼32 pN for 2G-HQ; see the dotted histogram in Figure 8, top panel), the populations formed without transcription or with deaza transcription (GQ/GT) show rupture forces of ∼25 ± 2 pN (see dotted histograms for deconvoluted GQ populations). This difference is statistically significant at a confidence level of 95% by the Analysis of variance (ANOVA) analyses, firmly demonstrating that HQ species are mechanically more stable than the GQ species. As the unfolding forces of both GQ and HQ are higher than the stall force of known RNAP species (35,36,38), the increased mechanical stability in HQ suggests it may serve as a more effective mechanical block to the RNAPs.

Using Jarzynski non-equilibrium theorem, we evaluated the thermodynamic stability of HQ versus GQ species based on the unfolding work of each species (16,25). After deconvoluting 2G-HQ, 3G-HQ and GQ species as described in literature (27) (see dotted histograms in Figure 8), we retrieved their respective change in free energy of unfolding (Δ*G*_unfold_) as 5.5 ± 0.1(0.4), 6.8 ± 0.3(−0.6) and 9.5 ± 0.3(0.8) kcal/mol [values in parentheses show the bias in the Δ*G*_unfold_ estimation (39)]. It is interesting that the GQ species show higher thermodynamic stabilities than HQ, which could be attributed to the increased entropic penalty for the formation of intermolecular structures in HQ with respect to intramolecular structures in GQ.

#### Transition kinetics of the HQ and GQ species

**Figure 9.**
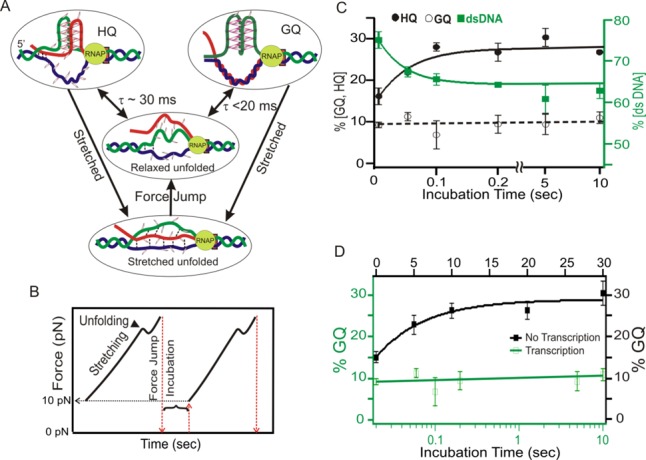
(**A**) Schematic diagram for different G-rich structures during the force jump experiment. Dotted lines represent possible Watson-Crick (WC) or Hoogsteen bonding. (**B**) Profile of force change during the FPP experiment. (**C**) The percent formation of HQ, GQ+GT and dsDNA for different incubation period during stalled transcription. (**D**) Comparison of the percent formation of the GQ+GT species with (green) and without (black) transcription. For clarity, the kinetics data for regular transcription are displayed with a semi log plot.

To investigate the temporal effect on the population dynamics of the GQ and HQ species, we used the FPP method (8,23). First, we unfolded all structures by mechanically stretching the nucleic acid construct up to 45 pN, followed by a quick relaxation to 0 pN within 20 ms. After a specific incubation time, we probed the folded structures by next round of force ramping that started at 10 pN by another force jump. Folding of a species was revealed by the rupture event while the identity of the species was determined by the change in contour length as a result of its unfolding (see Supplementary Figure S8). After plotting the major species of GQ and HQ (2G-HQ and 3G-HQ) versus the incubation time at 0 pN, we found that the percentage population of DNA GQ (∼10%) reached a steady state within 20 ms while the formation of HQs followed a single-exponential kinetics with a time constant of 30 ± 20 ms (Figure 9A). Comparison of the transition kinetics among HQ, GQ, and dsDNA species (Figure 9C) reveals that the formation of the HQ is at the expense of dsDNA until a steady state is reached at 100 ms. This suggested that the presence of a proximal RNA strand helps to convert the G/C-rich duplex DNA to the HQ within 100 ms. Such a fast kinetics suggested that co-transcriptional folding of HQ and GQ is feasible even without the pausing of RNAP during transcription. With a transcription rate of ∼10 bp/s (40), the folding and equilibrium of HQ and GQ could be accomplished as soon as the quadruplex hosting sequence is transcribed given that participating DNA and RNA strands remain single stranded.

Interestingly, the folding of DNA GQ in the absence of transcription was much slower (150 ± 60 ms; Figure 9D). It is possible that negative superhelicity in the wake of a transcription bubble may speed up the DNA GQ formation. However, since the superhelicity of the DNA template quickly reaches equilibrium by the free rotation of the stalled RNAP in our experiments, the superhelicity effect is not likely. An alternative explanation is that the presence of RNAP may facilitate the folding of DNA GQ. Given the fact that the stalled RNAP is located away from the G-core by ∼5 nm, this scenario is not likely either. Instead, we propose a catalytic role of nascent RNA for the GQ formation. We surmise that RNA could serve as a template to bring DNA guanine residues together, which reduces the entropic penalty to form the GQs. Another scenario could be that in the presence of an RNA transcript, the hybridization of complementary DNA strands is inhibited due to the formation of the R-loop, which leaves the single-stranded G-rich strand more available for the DNA GQ to fold.

We have shown here that mechanical stability of either GQ or HQ can withstand the stall force of known RNAPs (Figure 8). This implies a possible synergistic effect for GQ and HQ to serve as mechanical blockers to transcription. The synergistic effect is supported at the molecular level that nascent RNA transcripts serve as catalysts for the formation of GQ (Figure 9) while converting more dsDNA into HQ and GQ species (Table 1). Therefore, the potential biological functions of HQ lie in two folds. First, HQ may itself stall the transcription. Second, HQ could kinetically or thermodynamically change the population of GQ, which is a well-known modulator for various biological processes.

### Comparison between the SMSA and ensemble experiments

Our recent ensemble experiments have shown that during the first round of transcription, R-loop, instead of HQ, is formed (19). The formation of R-loop may be accompanied by a GQ folded in the non-template (G-rich) strand. To test this hypothesis using mechanical unfolding experiments, we prepared a construct that contained a stall site upstream of the G-core. Only adenine and guanine bases were present between the TSS and the stall site (see construct 2 in Supplementary Figure S3). The T7 RNAP was initially stalled by a supply of those two NTPs. The construct also contained a second stall site at the 15th nucleotide downstream of the G-core. Resuming the transcription of the RNAP stalled at the first site using three NTPs (ATP, GTP and UTP) would allow only one RNAP to pass the G-core and stall at the second site. Mechanical unfolding of this construct showed that folded species were identical (Supplementary Figure S7) with those in Figure 6. Interestingly, during the first few *F*–*X* curves, the ∼6-nm species (DNA GQ) was observed most often, which is consistent with the observation that HQ was not formed during the first round of transcription (19).

Our ensemble data also showed that during the second round of transcription, not only DNA GQ was unfolded by RNAP, but the RNA–DNA hybrid duplex (R-loop) was also displaced by the polymerase so that both single-stranded non-template (G-rich) DNA and previous RNA transcript were available for the HQ formation (19). This situation was well mimicked in our SMSA method in which all folded structures were first unfolded by mechanical stretching. In addition, mechanical tension is expected to weaken the Watson–Crick pairs (41) in the DNA or the hybrid DNA/RNA duplex. Therefore, the refolding of the species at the lower force in the single-molecule approach resembled the second pass of RNAP during ensemble experiments. The observation of HQ formation during subsequent *F–X* curves again supported the results from our ensemble experiments.

Subtle difference existed between the single molecule assay and the ensemble experiments. In the latter case, the DNA template strand was no longer available for population equilibrium as it was hybridized with the new RNA transcript during the second round of transcription. However, in the mechanical unfolding experiments, this strand was available for the population dynamics under mechanical tension. Such a subtle difference implied that amount of HQ formation might be underestimated in single-molecule experiments (26.7%) compared to ensemble assays.

Limitations exist for this SMSA. As T7 RNAP is stalled downstream of the G-core, it may have different conformation compared to the active enzyme during transcription, which may lead to different formation kinetics of GQ/HQ. In addition, small uncertainty exists in the estimation of folded population as it is difficult to determine the exact position of the stalled RNAP.

## CONCLUSION

Using ensemble experiments and an SMSA, we identified a population mixture of GQ and HQ in a natural G-rich sequence downstream of many TSSs in a T7 transcription model. We revealed that HQ predominated over GQ in population as well as in mechanical stability, which implies that HQ can serve as a more effective mechanical block for transcription. The fact that RNA transcripts catalyzed the folding of GQ while converting more dsDNA into quadruplex species starts to suggest a synergistic effect of HQ and GQ on the transcriptional control. As our SMSA approach closely mimics the transcription processes, we anticipate that the method can be applied to investigate complex molecular population dynamics that involve non-canonical DNA and RNA structures during transcription.

## SUPPLEMENTARY DATA

Supplementary Data are available at NAR Online, including [1–5].

SUPPLEMENTARY DATA
