# Supplementary material for: Nascent RNA transcripts facilitate the formation of G-quadruplexes
Source: Nucleic Acids Res. 2014 May 14;42(11):7236–46. doi: 10.1093/nar/gku416 (PMC4066803; doi:10.1093/nar/gku416)
Supplement: SUPPLEMENTARY DATA [file supp_42_11_7236__index.html]

Nascent RNA transcripts facilitate the formation of G-quadruplexes — SUPPLEMENTARY DATA 

# Nascent RNA transcripts facilitate the formation of G-quadruplexes

## SUPPLEMENTARY DATA

**Files in this Data Supplement:**

- SUPPLEMENTARY DATA
