# Supplementary material for: Nascent RNA transcripts facilitate the formation of G-quadruplexes
Source: Nucleic Acids Res. 2014 May 14;42(11):7236–46. doi: 10.1093/nar/gku416 (PMC4066803; doi:10.1093/nar/gku416)
Supplement: SUPPLEMENTARY DATA [file supp_gku416_nar-03724-f-2013-File002.docx]

**Supporting Information**

**Nascent RNA Transcripts Facilitate the Formation of G-quadruplexes**

Prakash Shrestha^1,#^, Shan Xiao^2,#^, Soma Dhakal^1^, Zheng Tan^2,^*, Hanbin Mao^1,^*

#These authors contribute equally.

1. Department of Chemistry and Biochemistry and School of Biomedical Sciences, Kent State University, Kent, OH, 44242. USA.

2. State Key Laboratory of Biomembrane and Membrane Biotechnology, Institute of Zoology, Chinese Academy of Sciences, Beijing 100101, P. R. China.

*Corresponding authors, z.tan@ioz.ac.cn, hmao@kent.edu

**Detection of HQ formation in synthetic oligonucleotides**

DNA:RNA heteroduplex was prepared and subjected to native gel electrophoresis or Circular Dichroism (CD) spectroscopic analysis as described,([1](#_ENREF_1)) except that 8 mM MgCl_2_ was used and the PEG was replaced by 30% DMSO.

**Calculation of Percent Formation of G-triplex, G-quadruplex, and DNA/RNA hybrid G-quadruplex structures**

To estimate the percent formation of different species in the control experiments (Figure *S4-S6*), we took the ratio of *F-X* curves that contain 2-6 nm Δ*L* features *vs* all *F-X* curves. The overall *F-X* curves under specific conditions are shown at the top of the bar diagrams (Figures*S5 and S6*).

The population at Δ*L* ~ 4 nm corresponds to the involvement of 14 nucleotides (see equation S1) in a structure, which could be either partially folded intramolecular DNA G-triplex or three G tracts participated in the DNA/RNA hybrid G-quadruplex (HQ). To find the percentage population of each structure, we first determined G-triplex population using the population ratio of G-quadruplex and G-triplex structures observed in the deaza-transcription, in which HQ is not expected to form. The HQ population was then estimated from the rest of the 4 nm Δ*L* population.

**Expected Change in Contour Length**

Assuming *x* is the end-to-end distance (distance between the two termini of a folded structure); the expected change in contour length (∆*L*) as a result of its unfolding can be calculated with the following equation,([2](#_ENREF_2),[3](#_ENREF_3))

Δ*L* = *N* × *L*_bp_ - *x* ……… ……. (*S1*)

Where *N* and *L*_bp_ are the number of base pairs involved in the structure and the contour length per nucleotide base pair, respectively. Here, *L*_bp_= 0.34 nm was used (because the fact that duplex DNA is obtained after unfolding, *L*_bp_ is equivalent to the contour length per base pair, 0.34 nm).([4](#_ENREF_4)) To calculate the expected ∆*L* of the G-quadruplex structures in agiven sequence, a valueof *x* = 1.0 (± 0.1) nm was taken from reported NMR structures of the hybrid-1 type quadruplex.([5](#_ENREF_5))

Four different folded structures equivalent to the following number of nucleotides were observed.Forintramolecular G-quadruplex structure (*N* = 19 nucleotides), equation *S1* gives an expected ∆*L* of5.5 (± 0.1) nm. Similarly, ∆*L* for all possible structures were calculated according to the number of nucleotides of the template DNA strand that may be involved in the folding. DNA/RNA (3G+1G) hybrid G-quadruplex and intramolecular DNA G-triplex structures with *N* = 14 give an expected ∆*L* of 3.8 (± 0.1) nm and DNA/RNA (2G+2G) hybrid G-quadruplex has an expected ∆*L* of2.0 (± 0.1) nm.

**Figure*S1*.**HQ formation between the (G_4_A)_4_ G-core in synthetic DNA and RNA oligonucleotides. (A) The RNA annealed with DNA1 or DNA2 was subjected to a digestion with RNase A or H before being resolved on a native gel. A retarded migration of the RNAs in lanes 3 and 4 in comparison with that of the RNA in lanes 6 and 7 in indicates a formation of HQ. (B) CD spectra of RNase H digested RNA + DNA1, RNA + DNA2, and RNase H.


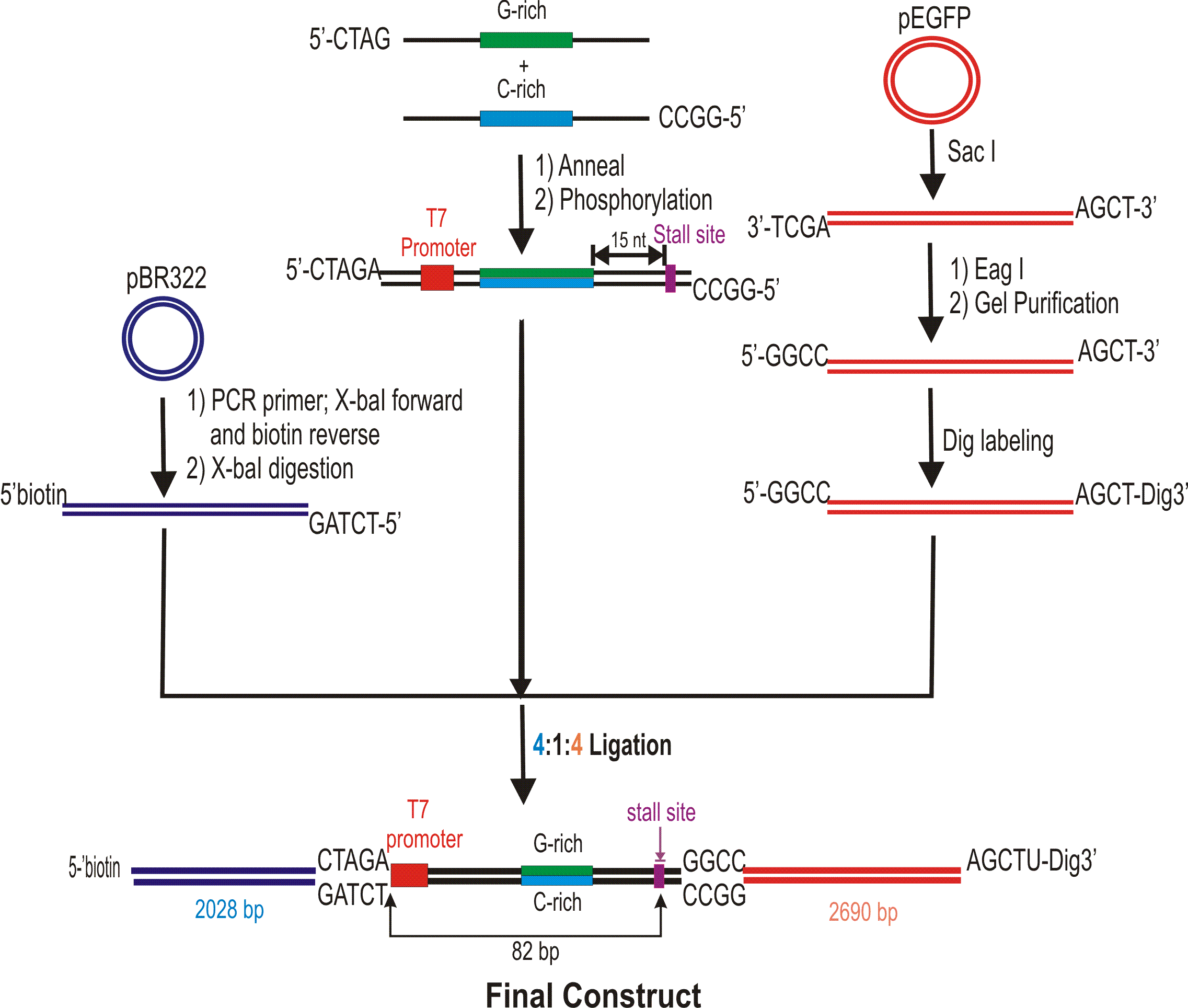


**Figure*S2*.**Preparation strategy of the DNA construct used for the mechanical unfolding and refolding during transcription. The 2690 bp handle was prepared from pEGFP plasmid by Sac I digestion, followed by Eag I digestion. The digested handle was labeled with digoxigenin at the 3' overhang using terminal transferase (NEB) and purified by agarose gel. The 2028 bp handle was prepared by PCR using a pBR322 template with a biotinylated primer and another primer that contains an XbaI restriction site. The complementary oligos consisting sequence of interest were annealed and phosphorylated. The final construct was obtained by 3-piece (the digoxigenin labeled 2690 bp handle, the phosphorylated double stranded DNA fragment, and the biotinylated 2028 bp handle) ligation with 4:1:4 molar ratio.


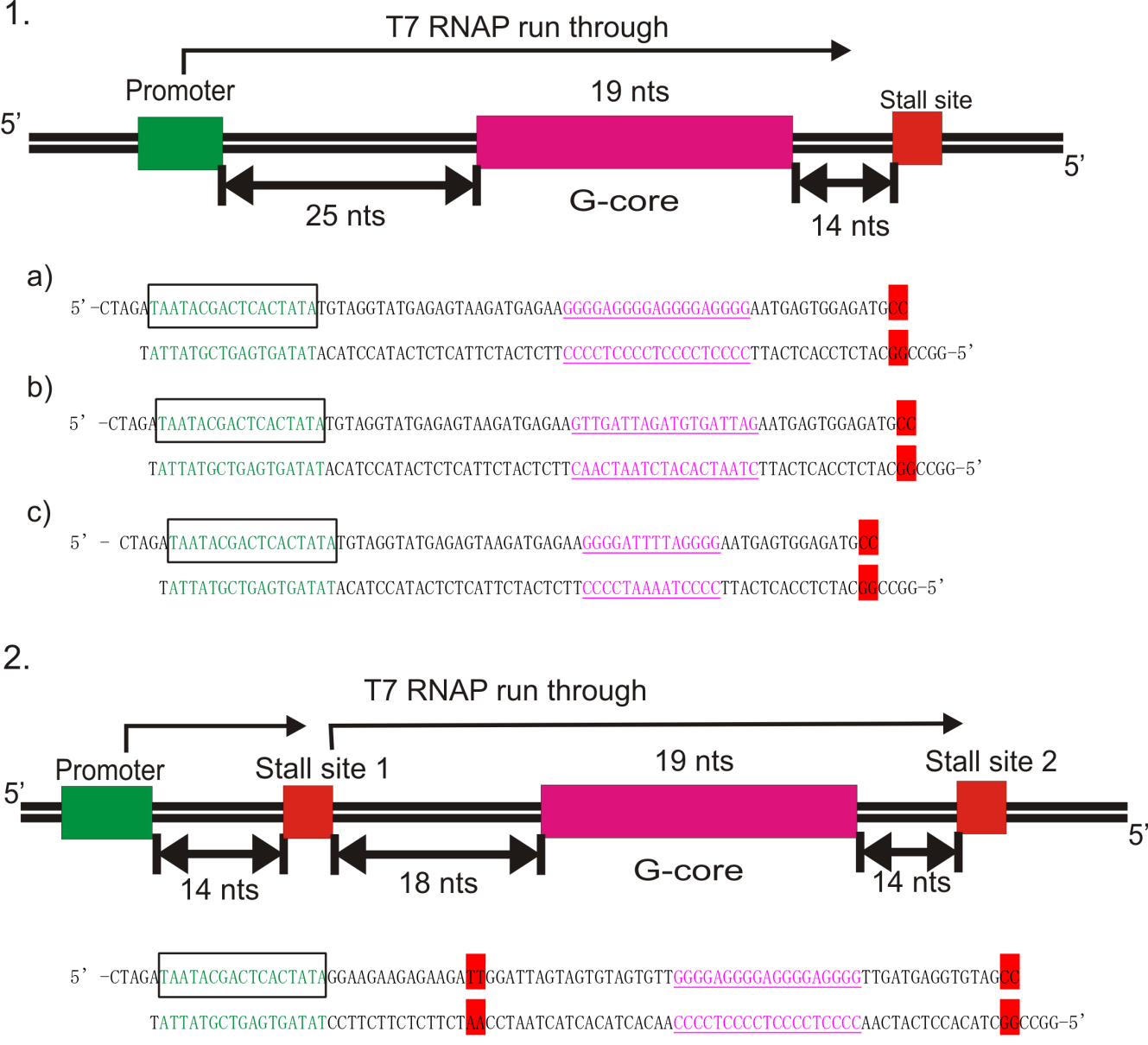


**Figure*S3*.**Schematics of the DNA construct for Single-Molecule Stalled-transcription Assay (SMSA). 1) DNA construct that contains a single stallsite (highlighted with red). a) GQ-forming construct (GQ-Construct) that contains a wild-type G-core sequence (pink).b) Control construct with a scrambled G-core sequence (Mutated-Control). c) Loop control construct: one G-tract is mutated to TTTT (bold) between the two G-tracts (Loop-Control). 2) DNA construct that contains two stallsites (highlighted with red). Boxed green sequences depict the T7 promoter.

**Figure*S4*.**Control experiments to confirm the formation of GQ species in the GQ forming construct (GQ-Construct). Percent formation of structures (featured curves) during transcription (a) and without transcription (b) in the single-molecule mechanical unfolding and refolding experiments in a 100 mM KCl buffer.

**Figure*S5*.**Comparison of the percentformation of GQ and HQ species in the stalled transcription assay in 100 mM KCl and 100 mM LiCl. The significantly reduced percent formation of structures in 100 mM LiCl (6.7 %) with respect to that in 100 mM KCl (42.7 %) supports that folded structures are GQ and HQ species. Numbers in parentheses indicate the F-X curves that contain folded structure *vs* total F-X curves.

**Figure*S6*.**Control experiments to show that consecutive G-tracts are necessary for HQ formation.See Figure*S3* for the sequence ofGQ-Construct, Mutated-Control,and Loop-Control. Rupture events with ∆*L* ranging from ~2-8 nmwere counted. Numbers in parentheses indicate the F-X curves that contain folded structure *vs* total F-X curves.

**
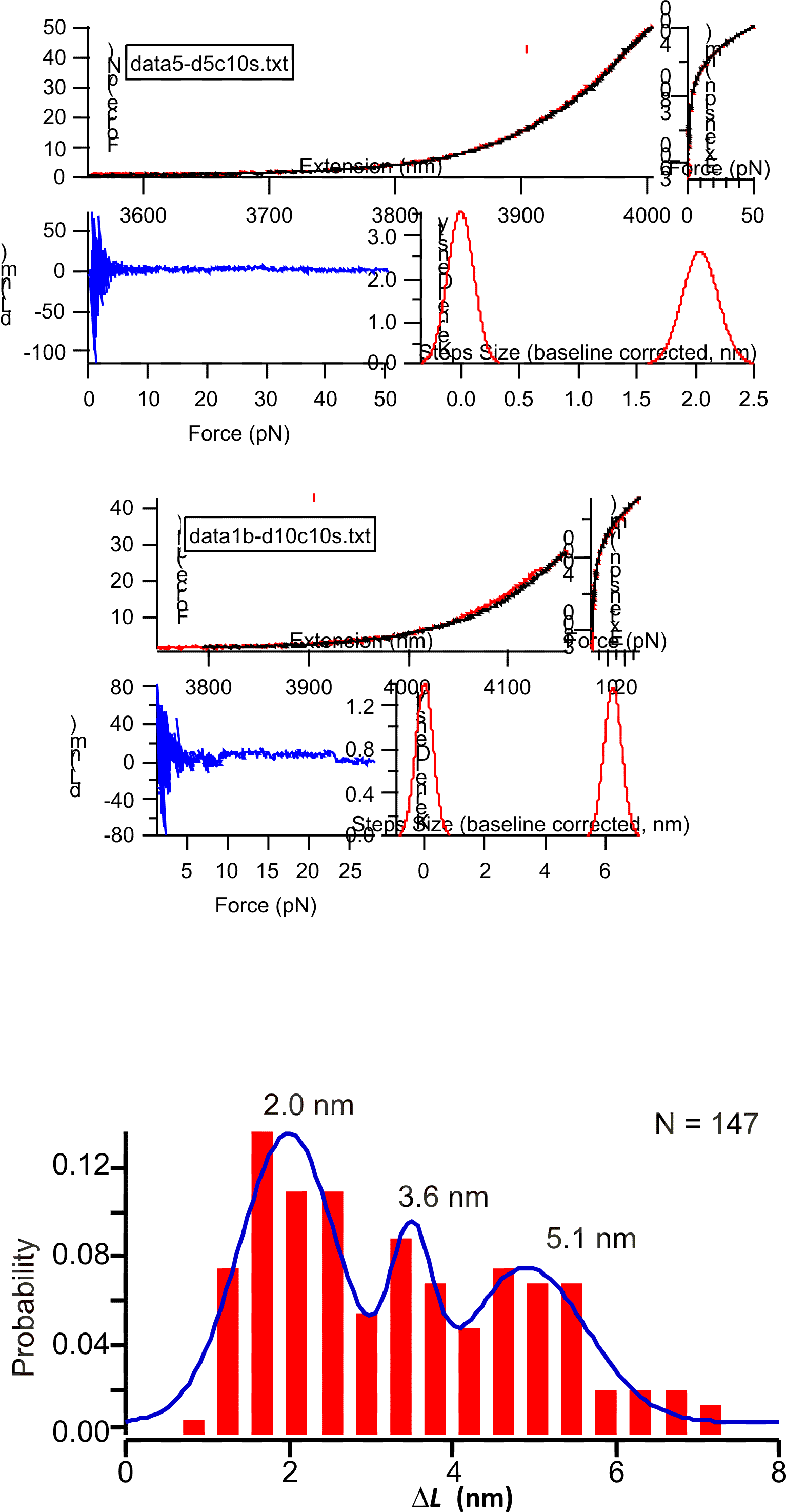
**

**Figure*S7***.Histogram of the change in contour length (Δ*L*) obtained from the single-molecule stalled-transcription assayon the DNA construct that contains two stall sites (see Figure*S3,* 2). The two stall-sitesdesign avoidstranscription due to multiple T7-RNAPs (see main text).


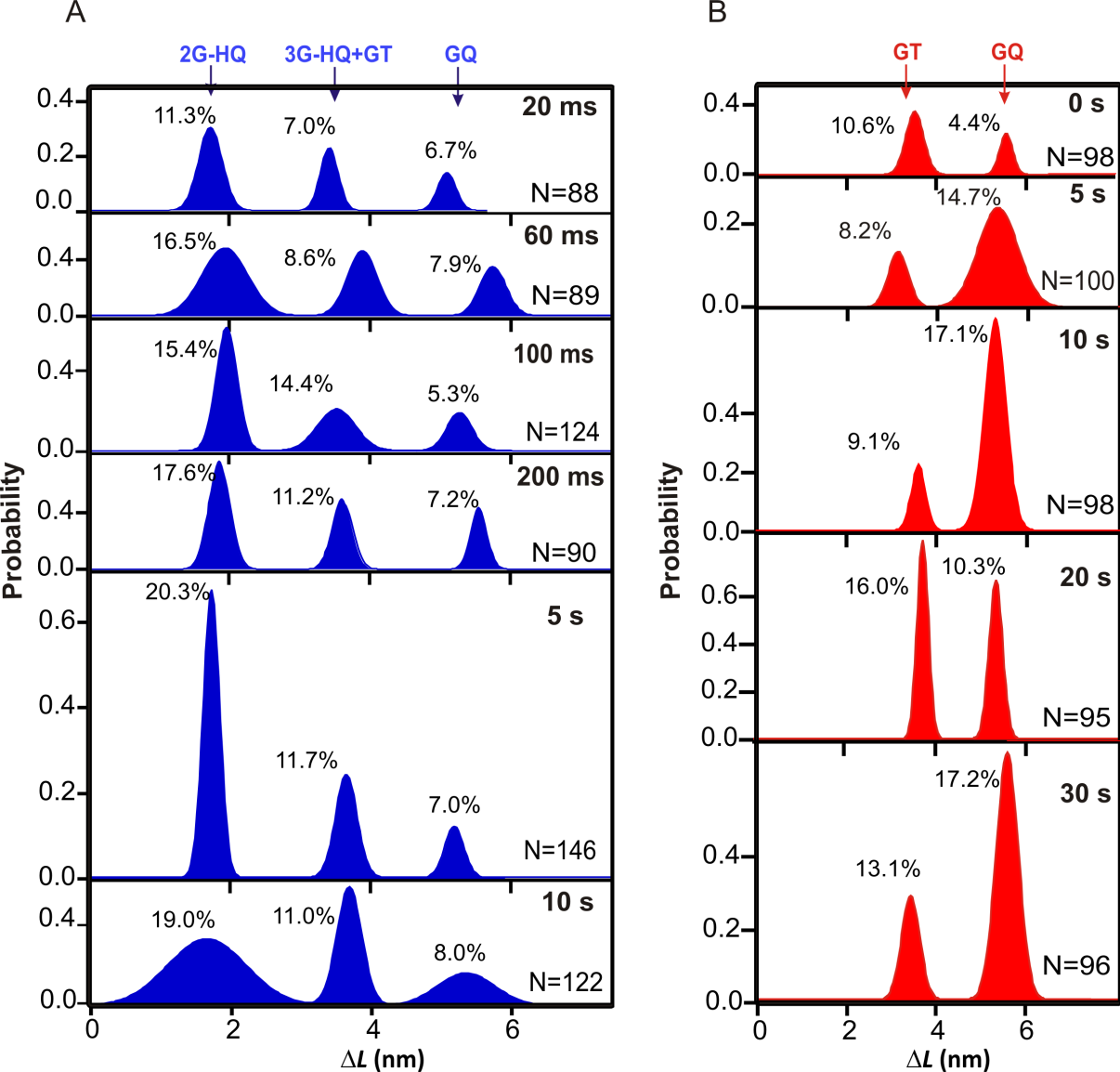


**Figure*S8*.**Histograms of the change in contour length (∆*L*) observed in A) transcription and B) without transcription at different incubation time. The histograms were plotted after the bootstrap analysis of ∆*L* measured from the force-extension curves (see Materials and Methods). The percentages shown are the absolute percentage of the corresponding populations (see *SI* text).

| **S.N.** | **Ensemble Gene ID** | **Associated Gene Name** | **Distance from TSS (bp)** | **S.N.** | **Ensemble Gene ID** | **Associated Gene Name** | **Distance from TSS (bp)** |
| --- | --- | --- | --- | --- | --- | --- | --- |
| 1 | [ENSG00000166341](http://asia.ensembl.org/Homo_sapiens/Gene/Summary?db=core;g=ENSG00000166341) | [DCHS1](http://asia.ensembl.org/Homo_sapiens/Gene/Summary?db=core;g=ENSG00000166341) | 10 | 19 | [ENSG00000204220](http://asia.ensembl.org/Homo_sapiens/Gene/Summary?db=core;g=ENSG00000204220) | [PFDN6](http://asia.ensembl.org/Homo_sapiens/Gene/Summary?db=core;g=ENSG00000204220) | 1554 |
| 2 | [ENSG00000101439](http://asia.ensembl.org/Homo_sapiens/Gene/Summary?db=core;g=ENSG00000101439) | [CST3](http://asia.ensembl.org/Homo_sapiens/Gene/Summary?db=core;g=ENSG00000101439) | 140 | 20 | [ENSG00000206283](http://asia.ensembl.org/Homo_sapiens/Gene/Summary?db=core;g=ENSG00000206283) | [PFDN6](http://asia.ensembl.org/Homo_sapiens/Gene/Summary?db=core;g=ENSG00000206283) | 1554 |
| 3 | [ENSG00000204540](http://asia.ensembl.org/Homo_sapiens/Gene/Summary?db=core;g=ENSG00000204540) | [PSORS1C1](http://asia.ensembl.org/Homo_sapiens/Gene/Summary?db=core;g=ENSG00000204540) | 181 | 21 | [ENSG00000224782](http://asia.ensembl.org/Homo_sapiens/Gene/Summary?db=core;g=ENSG00000224782) | [PFDN6](http://asia.ensembl.org/Homo_sapiens/Gene/Summary?db=core;g=ENSG00000224782) | 1554 |
| 4 | [ENSG00000231094](http://asia.ensembl.org/Homo_sapiens/Gene/Summary?db=core;g=ENSG00000231094) | [PSORS1C1](http://asia.ensembl.org/Homo_sapiens/Gene/Summary?db=core;g=ENSG00000231094) | 181 | 22 | [ENSG00000235692](http://asia.ensembl.org/Homo_sapiens/Gene/Summary?db=core;g=ENSG00000235692) | [PFDN6](http://asia.ensembl.org/Homo_sapiens/Gene/Summary?db=core;g=ENSG00000235692) | 1554 |
| 5 | [ENSG00000233439](http://asia.ensembl.org/Homo_sapiens/Gene/Summary?db=core;g=ENSG00000233439) | [PSORS1C1](http://asia.ensembl.org/Homo_sapiens/Gene/Summary?db=core;g=ENSG00000233439) | 181 | 23 | [ENSG00000237335](http://asia.ensembl.org/Homo_sapiens/Gene/Summary?db=core;g=ENSG00000237335) | [PFDN6](http://asia.ensembl.org/Homo_sapiens/Gene/Summary?db=core;g=ENSG00000237335) | 1554 |
| 6 | [ENSG00000233734](http://asia.ensembl.org/Homo_sapiens/Gene/Summary?db=core;g=ENSG00000233734) | [PSORS1C1](http://asia.ensembl.org/Homo_sapiens/Gene/Summary?db=core;g=ENSG00000233734) | 181 | 24 | [ENSG00000185087](http://asia.ensembl.org/Homo_sapiens/Gene/Summary?db=core;g=ENSG00000185087) | [FAM169B](http://asia.ensembl.org/Homo_sapiens/Gene/Summary?db=core;g=ENSG00000185087) | 2903 |
| 7 | [ENSG00000235487](http://asia.ensembl.org/Homo_sapiens/Gene/Summary?db=core;g=ENSG00000235487) | [PSORS1C1](http://asia.ensembl.org/Homo_sapiens/Gene/Summary?db=core;g=ENSG00000235487) | 181 | 25 | [ENSG00000144230](http://asia.ensembl.org/Homo_sapiens/Gene/Summary?db=core;g=ENSG00000144230) | [GPR17](http://asia.ensembl.org/Homo_sapiens/Gene/Summary?db=core;g=ENSG00000144230) | 3086 |
| 8 | [ENSG00000137094](http://asia.ensembl.org/Homo_sapiens/Gene/Summary?db=core;g=ENSG00000137094) | [DNAJB5](http://asia.ensembl.org/Homo_sapiens/Gene/Summary?db=core;g=ENSG00000137094) | 388 | 26 | [ENSG00000213199](http://asia.ensembl.org/Homo_sapiens/Gene/Summary?db=core;g=ENSG00000213199) | [ASIC3](http://asia.ensembl.org/Homo_sapiens/Gene/Summary?db=core;g=ENSG00000213199) | 3282 |
| 9 | [ENSG00000019485](http://asia.ensembl.org/Homo_sapiens/Gene/Summary?db=core;g=ENSG00000019485) | [PRDM11](http://asia.ensembl.org/Homo_sapiens/Gene/Summary?db=core;g=ENSG00000019485) | 580 | 27 | [ENSG00000141524](http://asia.ensembl.org/Homo_sapiens/Gene/Summary?db=core;g=ENSG00000141524) | [TMC6](http://asia.ensembl.org/Homo_sapiens/Gene/Summary?db=core;g=ENSG00000141524) | 4056 |
| 10 | [ENSG00000186471](http://asia.ensembl.org/Homo_sapiens/Gene/Summary?db=core;g=ENSG00000186471) | [AKAP14](http://asia.ensembl.org/Homo_sapiens/Gene/Summary?db=core;g=ENSG00000186471) | 582 | 28 | [LRG_118](http://asia.ensembl.org/Homo_sapiens/Gene/Summary?db=core;g=LRG_118) | [TMC6](http://asia.ensembl.org/Homo_sapiens/Gene/Summary?db=core;g=LRG_118) | 4056 |
| 11 | [ENSG00000167693](http://asia.ensembl.org/Homo_sapiens/Gene/Summary?db=core;g=ENSG00000167693) | [NXN](http://asia.ensembl.org/Homo_sapiens/Gene/Summary?db=core;g=ENSG00000167693) | 913 | 29 | [ENSG00000233493](http://asia.ensembl.org/Homo_sapiens/Gene/Summary?db=core;g=ENSG00000233493) | [TMEM238](http://asia.ensembl.org/Homo_sapiens/Gene/Summary?db=core;g=ENSG00000233493) | 4307 |
| 12 | [ENSG00000110887](http://asia.ensembl.org/Homo_sapiens/Gene/Summary?db=core;g=ENSG00000110887) | [DAO](http://asia.ensembl.org/Homo_sapiens/Gene/Summary?db=core;g=ENSG00000110887) | 994 | 30 | [ENSG00000106258](http://asia.ensembl.org/Homo_sapiens/Gene/Summary?db=core;g=ENSG00000106258) | [CYP3A5](http://asia.ensembl.org/Homo_sapiens/Gene/Summary?db=core;g=ENSG00000106258) | 4330 |
| 13 | [ENSG00000174403](http://asia.ensembl.org/Homo_sapiens/Gene/Summary?db=core;g=ENSG00000174403) | [C20orf166-AS1](http://asia.ensembl.org/Homo_sapiens/Gene/Summary?db=core;g=ENSG00000174403) | 996 | 31 | [ENSG00000166510](http://asia.ensembl.org/Homo_sapiens/Gene/Summary?db=core;g=ENSG00000166510) | [CCDC68](http://asia.ensembl.org/Homo_sapiens/Gene/Summary?db=core;g=ENSG00000166510) | 5516 |
| 14 | [ENSG00000259988](http://asia.ensembl.org/Homo_sapiens/Gene/Summary?db=core;g=ENSG00000259988) | [C20orf166-AS1](http://asia.ensembl.org/Homo_sapiens/Gene/Summary?db=core;g=ENSG00000259988) | 996 | 32 | [ENSG00000105642](http://asia.ensembl.org/Homo_sapiens/Gene/Summary?db=core;g=ENSG00000105642) | [KCNN1](http://asia.ensembl.org/Homo_sapiens/Gene/Summary?db=core;g=ENSG00000105642) | 5921 |
| 15 | [ENSG00000110887](http://asia.ensembl.org/Homo_sapiens/Gene/Summary?db=core;g=ENSG00000110887) | [DAO](http://asia.ensembl.org/Homo_sapiens/Gene/Summary?db=core;g=ENSG00000110887) | 1059 | 33 | [ENSG00000163082](http://asia.ensembl.org/Homo_sapiens/Gene/Summary?db=core;g=ENSG00000163082) | [SGPP2](http://asia.ensembl.org/Homo_sapiens/Gene/Summary?db=core;g=ENSG00000163082) | 8580 |
| 16 | [ENSG00000237345](http://asia.ensembl.org/Homo_sapiens/Gene/Summary?db=core;g=ENSG00000237345) | [RP11-344N17.11](http://asia.ensembl.org/Homo_sapiens/Gene/Summary?db=core;g=ENSG00000237345) | 1332 | 34 | [ENSG00000154153](http://asia.ensembl.org/Homo_sapiens/Gene/Summary?db=core;g=ENSG00000154153) | [FAM134B](http://asia.ensembl.org/Homo_sapiens/Gene/Summary?db=core;g=ENSG00000154153) | 9199 |
| 17 | [ENSG00000269683](http://asia.ensembl.org/Homo_sapiens/Gene/Summary?db=core;g=ENSG00000269683) | [RP11-344N17.11](http://asia.ensembl.org/Homo_sapiens/Gene/Summary?db=core;g=ENSG00000269683) | 1332 | 35 | [LRG_363](http://asia.ensembl.org/Homo_sapiens/Gene/Summary?db=core;g=LRG_363) | [FAM134B](http://asia.ensembl.org/Homo_sapiens/Gene/Summary?db=core;g=LRG_363) | 9199 |
| 18 | [ENSG00000173467](http://asia.ensembl.org/Homo_sapiens/Gene/Summary?db=core;g=ENSG00000173467) | [AGR3](http://asia.ensembl.org/Homo_sapiens/Gene/Summary?db=core;g=ENSG00000173467) | 1446 | 36 | [ENSG00000220291](http://asia.ensembl.org/Homo_sapiens/Gene/Summary?db=core;g=ENSG00000220291) | [RP3-455E7.1](http://asia.ensembl.org/Homo_sapiens/Gene/Summary?db=core;g=ENSG00000220291) | 9425 |

**Table *S1*.**Genes that contain the G-core sequence (5'-(G_4_A)_3_G_4_) downstream (within 10000 bp) of the transcription start site. Sequences of human genes were downloaded in thefasta format along with their IDs from the Ensemble genes database (release 74, GRCh37.p13) via the BioMart interface (http://www.ensembl.org/) by selecting known genes in the Gene type filter and Unspliced (Gene) in the Attribute/Sequences panel. The G-core was identified using the ZT-Find-PHQS-GUI.exe software supplied.([1](#_ENREF_1))

**Supporting References**

1. Zhang, J., Zheng, K., Xiao, S., Hao, Y. and Tan, Z. (2014) Mechanism and manipulation of DNA:RNA hybrid G-quadruplex formation in transcription of G-rich DNA. *J. Am. Chem. Soc.*, **136**, 1381–1390.

2. Dietz, H. and Rief, M. (2004) Exploring the energy landscape of GFP by single-molecule mechanical experiments. *Proc. Nat. Acad. Sci. USA*, **101**, 16192-16197.

3. Greenleaf, W.J., Frieda, K.L., Foster, D.A., Woodside, M.T. and Block, S.M. (2008) Direct observation of hierarchical folding in single riboswitch aptamers. *Science*, **319**, 630-633.

4. Sinden, R.R. (1995) *DNA Structure and Function*. Academic Press, San Diego, CA.

5. Dai, J., Punchihewa, C., Ambrus, A., Chen, D., Jones, R.A. and Yang, D. (2007) Structure of the intramolecular human telomeric G-quadruplex in potassium solution: a novel adenine triple formation. *Nucleic Acids Res.*, **35**, 2440-2450.
